# Supplementary material for: Splice-Junction-Based Mapping of Alternative Isoforms in the Human Proteome
Source: Cell Rep. Author manuscript; Available in PMC 2020 Jan 15. (PMC6961840; doi:10.1016/j.celrep.2019.11.026)
Supplement: 3 [file NIHMS1546469-supplement-3.zip › DF2/PXD006675/Aorta-42-O95359-ELEAMGLGTPSEAIEIR.pdf]

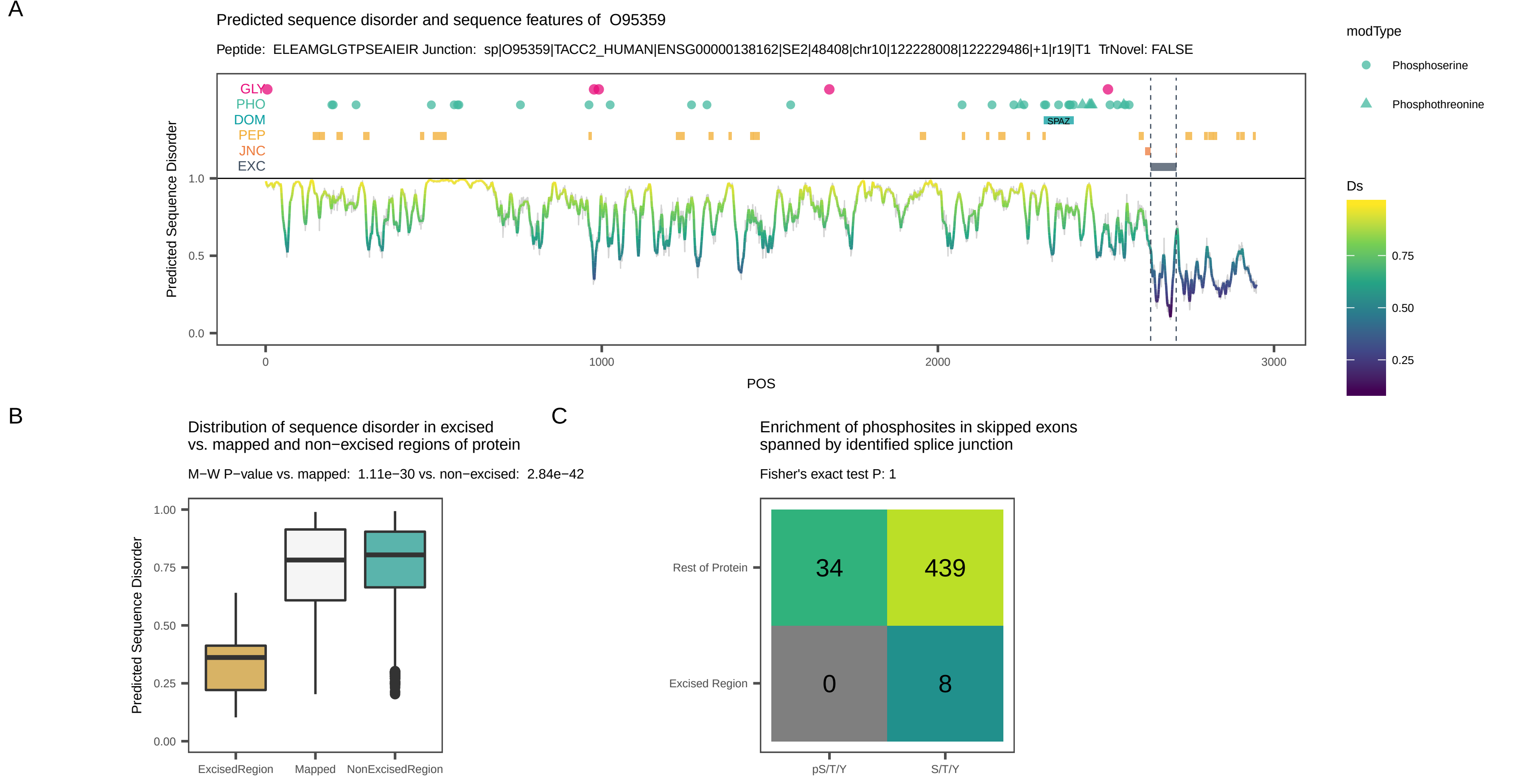
A
Predicted sequence disorder and sequence features of O95359
Peptide: ELEAMGLGTPSEAIEIR Junction: sp|O95359|TACC2_HUMAN|ENSG00000138162|SE2|48408|chr10|122228008|122229486|+1|r19|T1 TrNovel: FALSE
GLY
PHO
DOM
PEP
JNC
EXC
SPAZ
Predicted Sequence Disorder
1.0
0.5
0.0
0
1000
2000
3000
POS
modType
Phosphoserine
Phosphothreonine
Ds
0.75
0.50
0.25
B
Distribution of sequence disorder in excised
vs. mapped and non−excised regions of protein
M−W P−value vs. mapped: 1.11e−30 vs. non−excised: 2.84e−42
Predicted Sequence Disorder
1.00
0.75
0.50
0.25
0.00
ExcisedRegion
Mapped
NonExcisedRegion
C
Enrichment of phosphosites in skipped exons
spanned by identified splice junction
Fisher's exact test P: 1
Rest of Protein
Excised Region
34
439
0
8
pS/T/Y
S/T/Y
